# Ruminal paramphistomosis in cattle from northeastern Algeria: prevalence, parasite burdens and species identification

**DOI:** 10.1051/parasite/2014041

**Published:** 2014-10-06

**Authors:** Amal Titi, Abdeslam Mekroud, Mohamed el Hadi Chibat, Mehdi Boucheikhchoukh, Rima Zein-Eddine, Félicité F. Djuikwo-Teukeng, Philippe Vignoles, Daniel Rondelaud, Gilles Dreyfuss

**Affiliations:** 1 PADESCA Laboratory, Veterinary Science Institute, University Constantine 1 25100 El Khroub Algeria; 2 Department of Mathematics, Faculty of Exact Sciences, University Constantine 1 25100 El Khroub Algeria; 3 Department of Veterinary Science, University of El Tarf Algeria; 4 INSERM 1094, Faculties of Medicine and Pharmacy 87025 Limoges France; 5 Faculty of Health Sciences, Université des Montagnes BP 208 Banganté Cameroon

**Keywords:** Algeria, *Calicophoron daubneyi*, *C. microbothrium*, Cattle, Intensity of infection, Molecular identification, Paramphistomosis, Prevalence

## Abstract

Slaughterhouse samples were analysed over a two-year period (September 2010–August 2012) in Jijel (northeastern Algeria) in order to determine seasonal variations in the prevalence and intensity of bovine paramphistomosis in a Mediterranean climate and identify paramphistome species using molecular biology. In spring and summer, significantly higher prevalences and lower parasite burdens were noted in bull calves, thus indicating an effect of season on these parameters. In contrast, the differences among seasonal prevalences or among seasonal parasite burdens were not significant in the case of old cows. Eleven adult worms from the slaughterhouses of Jijel and three neighbouring departments (Constantine, El Tarf and Setif) were analysed using molecular markers for species identification. Two different species, *Calicophoron daubneyi* and *C. microbothrium*, were found. The presence of these two paramphistomids raises the question of their respective frequency in the definitive host and local intermediate hosts.

## Abstract (fr)

Des échantillons d’abattoir ont été analysés pendant deux années (septembre 2010 à août 2012) à Jijel (nord-est de l’Algérie) pour déterminer les variations saisonnières existant dans la prévalence et l’intensité de la paramphistomose bovine sous un climat méditerranéen et identifier l’espèce ou les espèces de paramphistomes par biologie moléculaire. Au printemps et en été, les prévalences sont significativement plus élevées et les charges parasitaires significativement plus faibles dans le cas des taurillons, ce qui indique un effet de la saison sur ces deux paramètres. Par contre, les différences entre les prévalences saisonnières ou les charges saisonnières en parasites adultes ne sont pas significatives dans le cas des vieilles vaches. Onze vers adultes provenant des abattoirs de Jijel et de trois départements avoisinants (Constantine, El Tarf, Sétif) ont été analysés à l’aide de marqueurs moléculaires pour l’identification des espèces. Deux espèces différentes, *Calicophoron daubneyi* et *C. microbothrium*, ont été trouvées. La présence de ces deux espèces de paramphistomes soulève la question de leur fréquence respective chez l’hôte définitif et leurs hôtes intermédiaires locaux.

## Introduction

Paramphistomosis (rumen fluke disease) is a trematodosis which affects domestic and wild ruminants. This infection is due to several genera such as *Calicophoron*, *Cotylophoron*, *Explanatum*, *Gigantocotyle* and *Paramphistomum* [22]. This parasitosis has a cosmopolitan distribution but its prevalence and intensity of infection may vary according to countries, climate and humid zones [22]. In temperate regions, paramphistomosis has a moderate impact on the livestock, whereas it causes greater losses in African countries because of the poor general state of ruminants [22, 33]. This parasitosis is known to have an insidious development and only very high infections of domestic ruminants cause a digestive symptomatology in adult animals. However, this pathology can be at the origin of major losses, especially characterised by diarrhoeas in young ruminants. For these reasons, a renewed interest for the study of this parasitosis throughout the world can be noted during the last few years. Several authors even consider paramphistomosis as an emerging or re-emerging disease [38].

In northeastern Algeria, little information on the occurrence of this parasitosis is available. According to Titi et al. [45], prevalence in local cattle ranged from 1.2% to 12.1% and its intensity varied from a mean of 87.5 to 984.1 worms per rumen according to surveyed slaughterhouses. However, these first findings were recorded over short periods of time (3–6 months per slaughterhouse [45]) so that the influence of local climate and, consequently, that of seasons were not analysed in the first report. A longer period of study (2 years, for example) was thus necessary to study the influence of these climatic factors. On the other hand, species identification was performed on adult worms using morphological and histological criteria so that *Calicophoron daubneyi* (Dinnik, 1962) Eduardo, 1983 [8, 9] was reported by Titi [43] and Titi et al. [45]. However, we wondered if *C. daubneyi* was the single paramphistome species in northeastern Algeria because several species of paramphistomes have already been reported, for example, in most European countries [35, 36]. In view of these first results, the following two questions arose: did the prevalence and intensity of bovine paramphistomosis show variations in relation to seasons in a Mediterranean climate? Was *C. daubneyi* the only paramphistome species in northeastern Algeria? To answer the first question, slaughterhouse samples were analysed for bovine paramphistomosis during a 24-month period in Jijel. The second question was answered by molecular biology, as this method can identify parasite species with little risk of error.

## Materials and methods

### Area of study

Worms were collected weekly over a 24-month period (September 2010–August 2012) at the slaughterhouse of Jijel. Located on mainly siliceous subsoil, this department (wilaya) forms part of the coastal band and its altitude is often less than 300 m. Its Mediterranean climate is sufficiently humid, with the mean monthly temperature varying from 8.4 °C to 27.7 °C and mean annual rainfall varying from 1103 to 1252 mm during the study period (Table 1). All cattle slaughtered (local breed, Guelmoise or Brown Atlas) originated from farms located within a 50-km radius around the slaughterhouse. These animals were reared under extensive conditions throughout the year in family structures (a few cattle per farm). Benzimidazole-type anthelmintics against intestinal and lung strongylids were mainly administered twice a year to cattle. In contrast, paramphistomosis was not treated.

**Table 1.** Monthly temperatures and rainfall in the department (wilaya) of Jijel during the two-year study (September 2010–August 2012).

Month	September 2010–August 2011		September 2011–August 2012	
	Temperature (°C)	Rainfall (mm)*	Temperature (°C)	Rainfall (mm)**
September	23.2	52.8	24.1	16.2
October	20.2	214.6	20.4	157.3
November	15.6	193.3	17.2	203.0
December	12.9	109.4	13.1	142.7
January	11.9	138.0	10.8	62.8
February	11.4	144.5	8.4	375.8
March	14.1	99.4	13.2	98.1
April	17.0	67.8	15.6	192.7
May	19.2	40.9	18.6	0.3
June	22.2	29.1	23.8	0
July	26.4	8.2	26.6	0.3
August	26.1	5.1	27.7	3.1

Annual rainfall: *1103.1 mm; **1252.3 mm.

### Parasitological investigations

A total of 1523 rumens from 1402 bull calves (<18 months in age) and 121 older cows (>5 years) were emptied and washed. Adult parasites collected from each rumen were placed in a polyethylene bag containing a saline solution (0.9% NaCl, 0.45% glucose). The parasites were transported to the laboratory at 37 ± 2 °C and immediately counted by two people.

Prevalence of infection was calculated using the ratio: number of infected animals/total number of cattle × 100, and was separately expressed for each category of infected animals (bull calves or cows) and each season (Tables 2 and 3). A χ^2^ test was used to determine the influence of season on these values. The intensity of paramphistome infection was the second parameter and was also expressed for each category of infected animals and each season (Tables 2 and 3). Normality of parasite burdens was analysed using the Shapiro-Wilk normality test [37]. As their distribution was not normal, the Kruskal-Wallis test was used to establish levels of statistical significance. All analyses were performed using XLSTAT software (http://www.xlstat.com/fr).

**Table 2.** Prevalence and intensity of bovine paramphistomosis in bull calves for each season during the two-year study.

Season	September 2010–August 2011		September 2011–August 2012	
	Number of bull calves studied (prevalence %)	Mean parasite burden ± *SD*	Number of bull calves studied (prevalence %)	Mean parasite burden ± *SD*
Autumn	253 (14.6)	160.9 ± 130.4	211 (13.7)	185.4 ± 133.5
Winter	176 (9.09)	178.1 ± 71.3	169 (8.2)	214.6 ± 151.8
Spring	185 (20.0)	99.1 ± 80.9	121 (23.1)	78.8 ± 104.3
Summer	169 (23.0)	101.3 ± 75.8	118 (16.1)	98.8 ± 74.8
Total	783 (14.6)	125.8 ± 99.9	619 (14.5)	139.4 ± 131.0

**Table 3.** Prevalence and intensity of bovine paramphistomosis in old cows for each season during the two-year study.

Season	September 2010–August 2011		September 2011–August 2012	
	Number of old cows studied (prevalence %)	Mean parasite burden ± *SD*	Number of old cows studied (prevalence %)	Mean parasite burden ± *SD*
Autumn	18 (33.3)	394.5 ± 329.3	11 (45.4)	295.6 ± 137.2
Winter	16 (25.0)	423.0 ± 148.3	8 (37.5)	594.3 ± 82.4
Spring	13 (69.2)	229.0 ± 79.4	20 (45.0)	234.2 ± 136.3
Summer	12 (66.6)	306.6 ± 260.1	23 (39.1)	208.4 ± 75.7
Total	59 (45.7)	313.8 ± 231.1	62 (41.9)	272.9 ± 165.8

**Figure 1.**
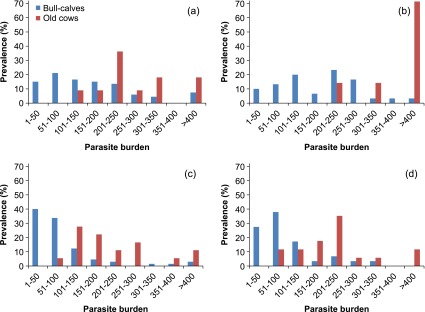
Numerical distribution of parasite burdens in relation to prevalence of bovine paramphistomosis for autumn (1a), winter (1b), spring (1c) and summer (1d). To build these graphs, the values obtained for corresponding seasons of both years were pooled.

Table 2 gives the prevalence and intensity of infection noted in bull calves for each season: autumn (September–November), winter (December–February), spring (March–May) and summer (June–August). A similar presentation is used for old cows in Table 3. To compare the distribution of parasite burdens noted for bull calves and cows in relation to season, individual values were classified into one of the following categories: 1–50, 51–100, 101–150, 151–200, 201–250, 251–300, 301–350, 351–400 and >400 worms per infected ruminant. The values recorded for each category during the corresponding seasons of both years were pooled and the results are shown in Figure 1.

### Molecular identification of paramphistomids

Eleven adult paramphistomes were collected from the rumens of cattle slaughtered in Jijel (3 worms) and in three other neighbouring abattoirs: Constantine (1 worm), El Tarf (4 worms) and Setif (3 worms), with a single worm per infected ruminant. After collection, parasites were washed with physiological saline solution (0.9% NaCl) and fixed in 70% ethanol until their use for molecular identification.

Total DNA was extracted from each worm using the DNeasy^®^ Tissue Kit (Qiagen) according to the manufacturer’s instructions. A Nanodrop ND-1000 spectrophotometer was used to quantify and check DNA purity. The internal transcribed spacer (ITS-2) fragment was amplified using the following primers: ITS2-F (5′-TGTGTCGATGAAGAGCGCAG-3′) and ITS2-R (5′-TGGTTAGTTTCTTTTCCTCCGC-3′) [16]. Amplification was performed in a 25-μL total reaction volume containing: 0.5 U Taq DNA polymerase (Qiagen), 5 μL 10× PCR buffer, 1 μL MgCl_2_ (1.5 mM), 0.8 μL dNTPs (0.2 mM) and 0.5 μL of each primer (20 ng/μL). A standard DNA concentration (500 ng/μL) was used for all PCR reactions. The PCR conditions were as follows: initial denaturation (10 min, 94 °C); 35 cycles, each comprising denaturation (60 s, 94 °C), annealing (90 s, 53 °C) and extension (60 s, 72 °C); final extension (10 min, 72 °C). PCR products were purified using MultiScreen^®^ HTS-HV Filter Plates (Merck Millipore) and Sephadex^®^ G-50 Superfine (Fisher Scientific), then sequenced using an ABI Prism BigDye^®^ Terminator v1.1 Cycle Sequencing Ready Reaction Kit (Applied Biosystems), and run on an ABI 377 automated sequencer.

The sequences obtained were used to perform BLAST searches [2] via the National Center for Biotechnology Information’s GenBank database in order to ensure that parasite and other potential contaminating sequences had not been obtained in error. Sequences were then corrected and translated into amino acid sequences using the invertebrate mitochondrial genetic code and inspected for stop codons using SeqScape v2.5. Nucleotide sequences were multiply aligned using CLUSTAL X [42]. A unique sequence was obtained for each individual.

The new sequences have been deposited in GenBank as LN610457, LN610458 and LN610459.

Measurements of overall haplotypes and nucleotide diversity were calculated using DnaSP v5 [24]. Pairwise genetic diversity was calculated using Tamura and Nei’s three-parameter model [40] and Mega v5. Phylogenetic analyses were conducted by maximum likelihood (ML), maximum parsimony (MP) and neighbour-joining (NJ) using Mega v5 [23]. This analysis was subjected to 2000 bootstraps as a means of testing the reliability of individual branches within the generated tree. *Paragonimus westermani* (Kerbert, 1878) Braun, 1899 [5, 20] (AB354218.1) was nominated as the outgroup. The following GenBank data: AY790883.1, *C. daubneyi*; GU735640.1, *Calicophoron microbothrium* (Fischoeder, 1901) Eduardo, 1983 [9, 11]; and HM026462.1, *Paramphistomum cervi* (Zeder, 1790) Fischoeder, 1901 [11, 47] were included in the analysis in order to examine the topology of the tree.

The nomenclature used to identify paramphistome species was that adopted by Jones [17].

## Results

### Prevalence of bovine paramphistomosis

Annual prevalence of infection in bull calves (Table 2) was 14.6% and 14.5% during the first and second years, respectively. Seasonal variations were noted, with the lowest percentages in winter (8.2–9.0%) and the highest in summer 2011 (23.0%) and spring 2012 (23.1%). Prevalence values recorded in spring and summer during the first year were significantly greater (χ^2^ = 14.62, *p* < 0.01) than those noted in autumn and winter. A similar finding was also noted for the second year (χ^2^ = 12.86, *p* < 0.01). In contrast, the differences between values noted during the 2 years for a given season were not significant, whatever the type of season. In spite of the lower number of old cows slaughtered in Jijel (Table 3), high annual prevalences were recorded: 45.7% during the first and 41.9% during the second year. The differences among seasonal percentages during the 2 years were not significant. Similar results were also obtained when values noted during the 2 years for a given season were compared.

Cows were significantly more infected than bull calves during the first (χ^2^ = 31.17, *p* < 0.001) and second years (χ^2^ = 29.92, *p* < 0.001). This finding was also noted for each season considered separately (data not shown).

### Parasite burdens

In bull calves (Table 2), the mean annual intensity recorded during the first and second years was 125.8 and 139.4 adult worms, respectively. Significant seasonal variations (first year: *H* = 16.68, *p* < 0.001; second year: *H* = 24.13, *p* < 0.001) were noted, with lower numbers of worms in spring and summer when the bull calves showed the highest prevalence values. In contrast, the differences between parasite burdens recorded for the corresponding seasons of both years were not significant, whatever the type of season. The mean annual intensity in old cows (Table 3) was clearly greater, with 313.8 and 272.9 adult worms counted during the first and second year, respectively. No significant differences among the four seasons for a given year or the corresponding seasons of both years were noted for this category of infected cattle.

When parasite burdens noted in old cows were compared with those from bull calves, the values were significantly greater in cows (first year: *H* = 33.42, *p* < 0.001; second year: *H* = 19.15, *p* < 0.001). Significant differences in relation to season were also noted for both springs, both summers and autumn 2010, while the other three differences were not significant (data not shown).

Figure 1 gives the numerical distribution of parasite burdens for both categories of infected ruminants in relation to season. In autumn (Fig. 1a) and winter (Fig. 1b), more than 200 paramphistomes were found in 31.8% (21/66) and 50% (15/30) of bull calves, respectively. Prevalences were lower in spring (Fig. 1c) and summer (Fig. 1d), as 9.2% (6/65) and 13.7% (8/58) of bull calves, respectively, contained more than 200 adult worms. In the case of old cows, the percentages were more variable. Burdens greater than 200 paramphistomes were noted in 44.4% (8/18) of cows in autumn, 100% (7/7) in winter, 44.4% (8/18) in spring and 58.8% (10/17) in summer.

### Molecular identification of paramphistomid species

A data matrix of 428 bp was used in the analysis of the nuclear ribosomal ITS-2. The sequences were found to have strong similarity. The analysed region contained nine variable sites (seven parsimony informative sites), with an overall nucleotide diversity (*π*) of 0.009. Six different haplotypes were identified, with observed haplotype diversity (Hd) of 0.80 across all the studied taxa. In the sample from El Tarf, only one haplotype was observed. In contrast, in the samples from Setif and Jijel, different haplotypes were observed and were closely related, differing from each other by one or two base substitutions. The mean evolutionary diversity for the entire population was low (*D* = 0.009) and the coefficient of evolutionary differentiation (Cd) was 2.46.

**Figure 2.**
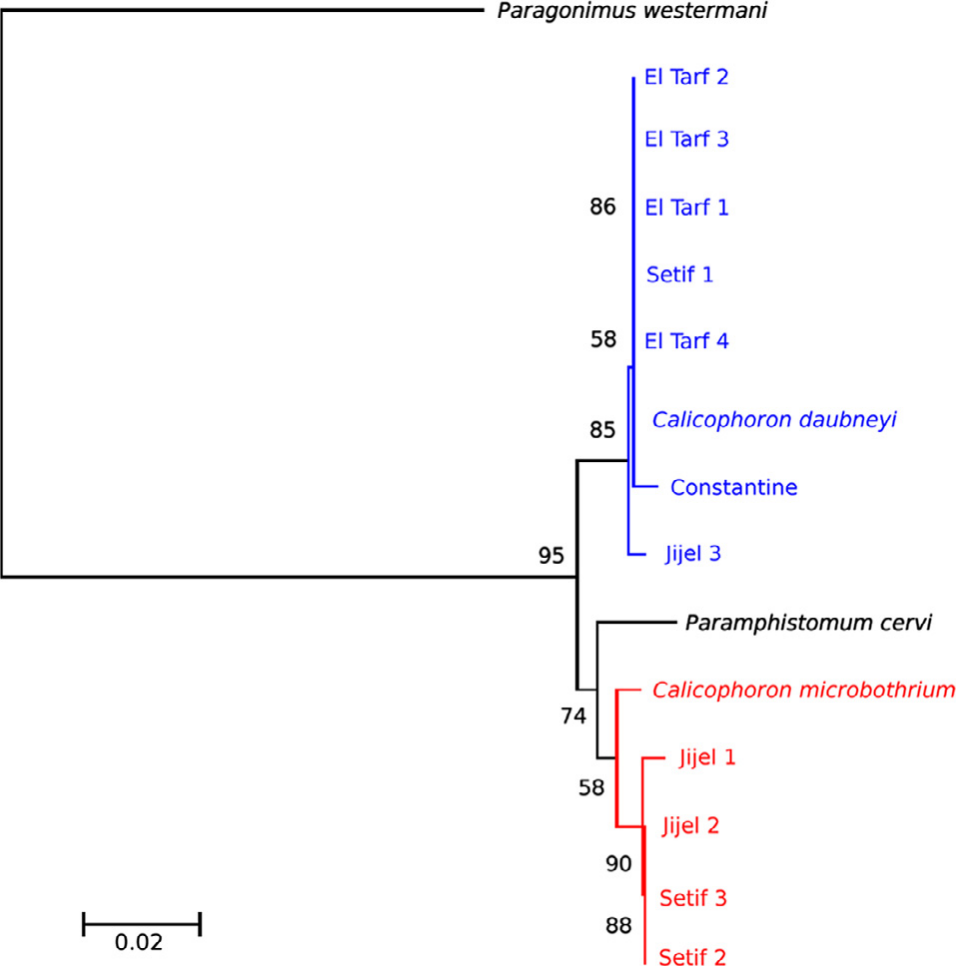
ITS-2 tree based on neighbour-joining estimates with *Paragonimus westermani* as the outgroup. In blue, clade 1; in red: clade 2. Scale bar indicates the number of substitutions per sequence position.

Reconstruction of the evolutionary relationships between haplotypes using ITS-2 is presented in Figure 2. The three tree-building algorithms were largely congruent, with high bootstrap support values of 89% for ML, 86% for MP and 90% for NJ among the different taxa. The studied haplotypes split into two clades. The first clade regroups *C. daubneyi* and seven worms from the four departments (Constantine, El Tarf, Jijel and Setif); within this clade, *C. daubneyi* and the worms from El Tarf and Setif share the same haplotype. The second clade regroups *P. cervi*, *C. microbothrium* and four worms from Jijel and Setif; within this group, the worms from Jijel and Setif form a sister group with *C. microbothrium.*

## Discussion

In bull calves slaughtered in Jijel, there was a significant effect of season on the prevalence and intensity of paramphistomosis. This seasonal effect is mainly due to the level of annual rainfall (1103–1252 mm, Table 1) and mild temperatures recorded in this coastal department, which creates a sufficiently humid and more temperate microclimate in spite of its Mediterranean type. This effect of season on the characteristics of cattle paramphistomoses in Jijel confirms the reports of different authors in temperate regions [3, 6, 7, 12, 39, 41] and subtropical or tropical countries [10, 15, 21, 34]. According to Hakalahti et al. [13], rainfall had a direct effect on the dynamics of parasite populations and, consequently, on disease transmission. As for fasciolosis [26, 31, 46], climatic conditions had a direct influence on the characteristics of *C. daubneyi* infection in the definitive and snail hosts of a given region. The significantly decreased prevalence noted for bull calves in autumn and winter might be due to the presence of numerous less than one-year-old individuals among the bull calves slaughtered during these periods. Owing to the death of numerous metacercariae encysted on vegetation during summer drying [27], most of these younger bull calves would not have enough time to become infected and/or harbour adult worms in their rumen before their slaughter. The progressive contamination of older bull calves (>12 months) during the next spring with metacercariae produced by the overwintering and/or spring snail generations [27, 28] and their progressive transformation into adults might explain the significantly lower parasite burdens noted in spring and summer, and the higher burdens during the next autumn and winter.

Contrary to bull calves, no significant seasonal effect on the characteristics of paramphistomosis was noted in old cows. This category of cattle was, however, more infected (41.9–45.7%) and contained high parasite burdens (a mean of 272–313 adult paramphistomes, Table 3). As the age of these slaughtered cows was more than 5 years, the most reliable explanation was an increasing accumulation of adult paramphistomes in their rumen during the past years in reason of repeated infections with metacercariae. However, another explanation, based on variability in the individual susceptibility of these cows to paramphistome infection, cannot be completely excluded. This last assumption is supported by the minimal and maximal individual burdens (84 and 1123 adult worms, respectively) found in the present study (data not shown). The high prevalence recorded for this category of cattle might be partly explained by the low number of these slaughtered cows (a total of 121 old cows instead of 1402 bull calves investigated during the two-year study).

The two paramphistome species, i.e. *C. daubneyi* and *C. microbothrium*, identified by molecular biology in the present study were already known in central Algeria [18, 19, 32]. As these two species were found in the coastal departments (El Tarf, Jijel) as well as on high plateaus of Atlas (Constantine, Setif; Fig. 2), their distribution is general in these four Algerian departments. The presence of both species in Jijel, for example, raises two fundamental questions. First, it is difficult to determine the importance of either paramphistomid in the definitive host because identification of all worms present in the rumen requires the use of PCR and, consequently, is expensive. Moreover, the possibility of cattle co-infections with both paramphistomids cannot be excluded. Secondly, the question of specific intermediate hosts (*Galba truncatula* Müller, 1774 [30] for *C. daubneyi* and *Bulinus truncatus* Audoin, 1827 [4] for *C. microbothrium*) merits close examination. Indeed, the presence of the bulinid in northeastern Algeria was not noted by Mekroud [27], Mekroud et al. [28] and Titi [45] during their field investigations. As the recruitment of slaughtered cattle was local (within a 50-km radius), it is necessary either to pursue investigations within these northeastern departments to find bulinid habitats, or to determine whether or not *C. microbothrium* uses another freshwater snail (another planorbid, for example) as a local intermediate host. Such examples of uncommon snail hosts have already been reported for *F. hepatica*, with *Anisus leucostoma* Millet, 1813 [29] and *Omphiscola glabra* Müller, 1774 [30] in central France [1] and *B. truncatus* in northern Tunisia [14].

In conclusion, bovine paramphistomosis existing in northeastern Algeria is dependent on seasons and is caused by at least two species: *C. daubneyi* and *C. microbothrium*. Further studies are still necessary (i) to determine the prevalence and intensity of infection caused by each paramphistome species in local cattle, and (ii) to specify the local intermediate host in the case of *C. microbothrium*.
